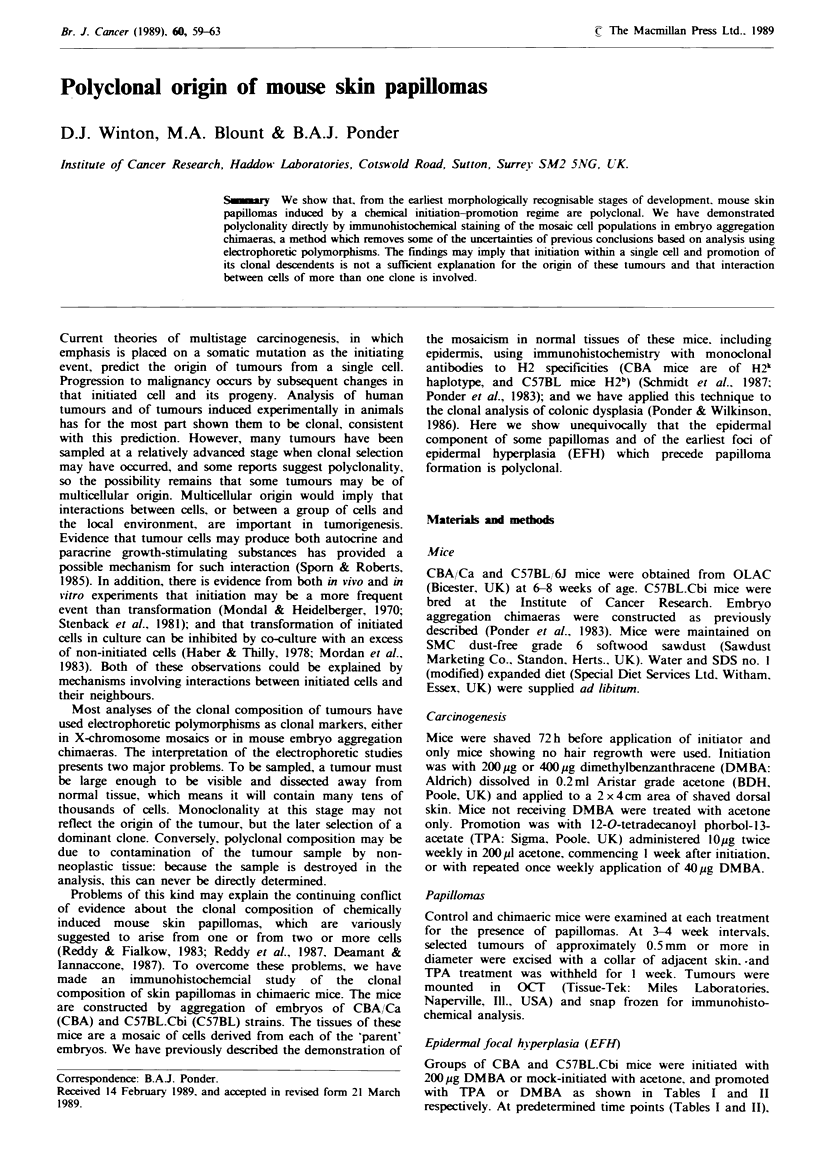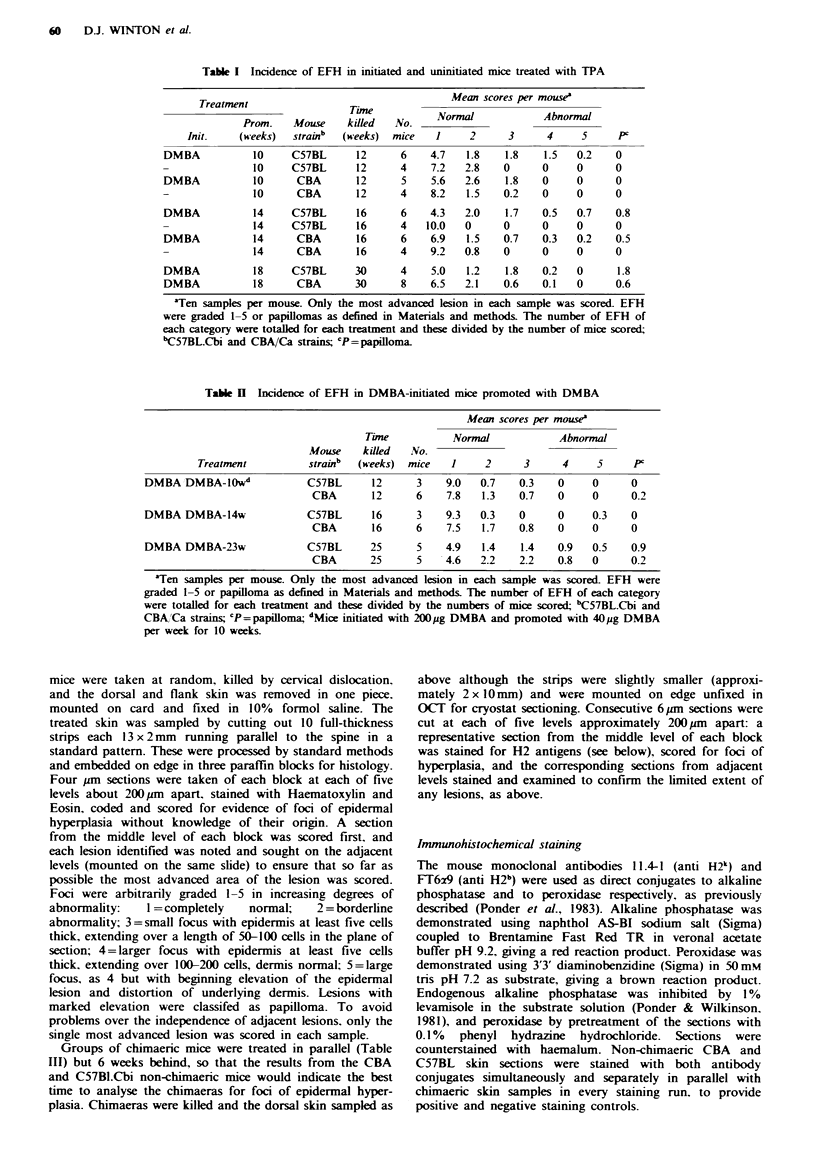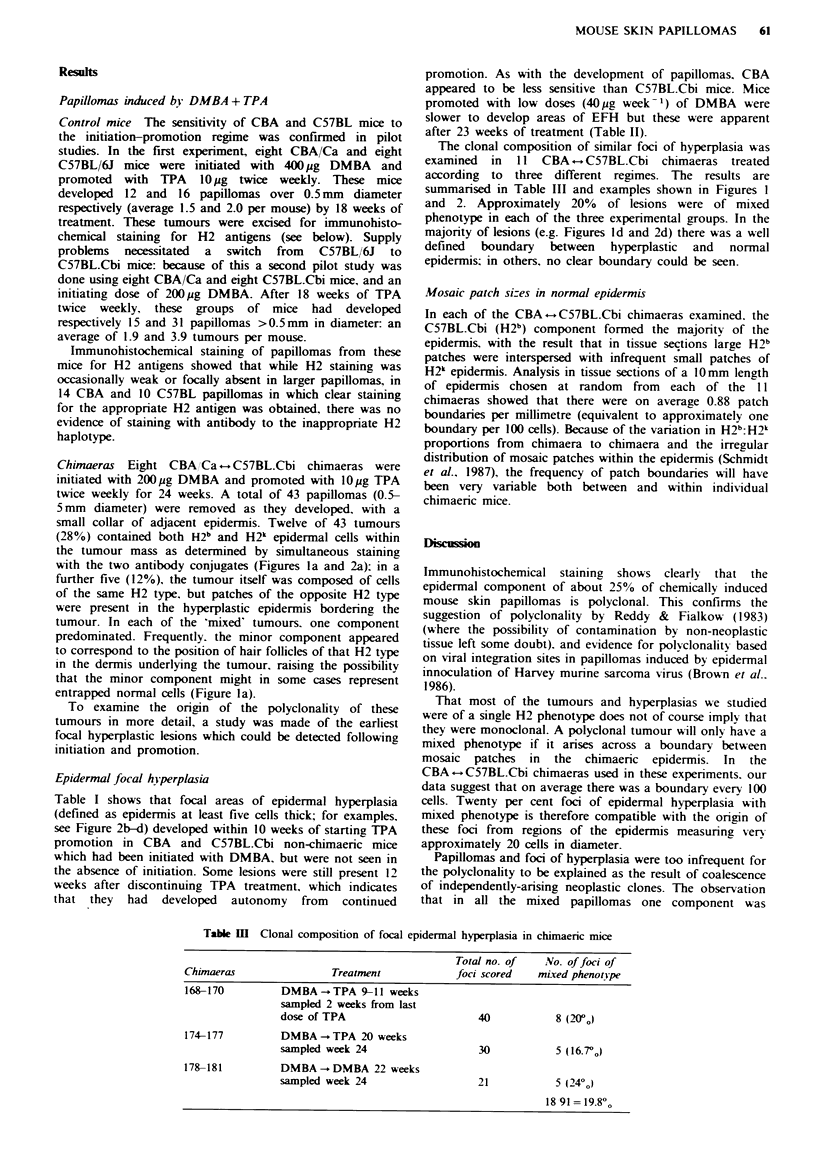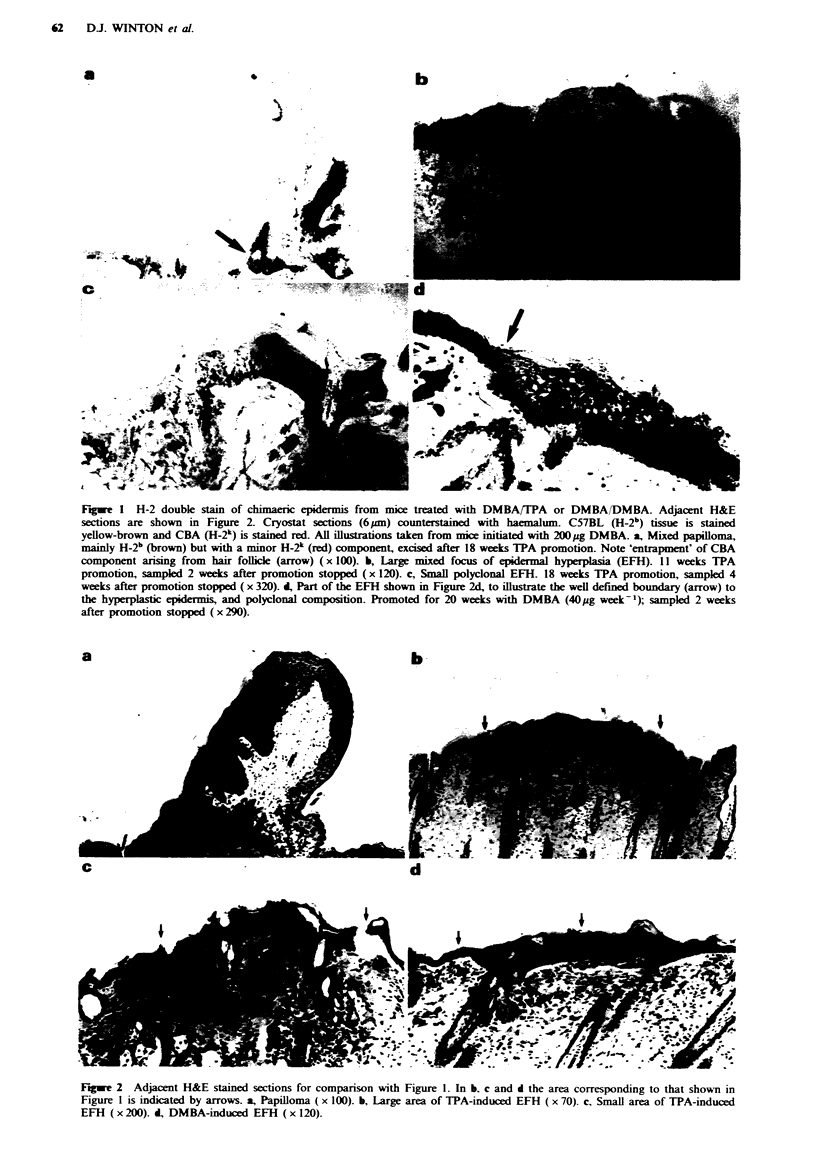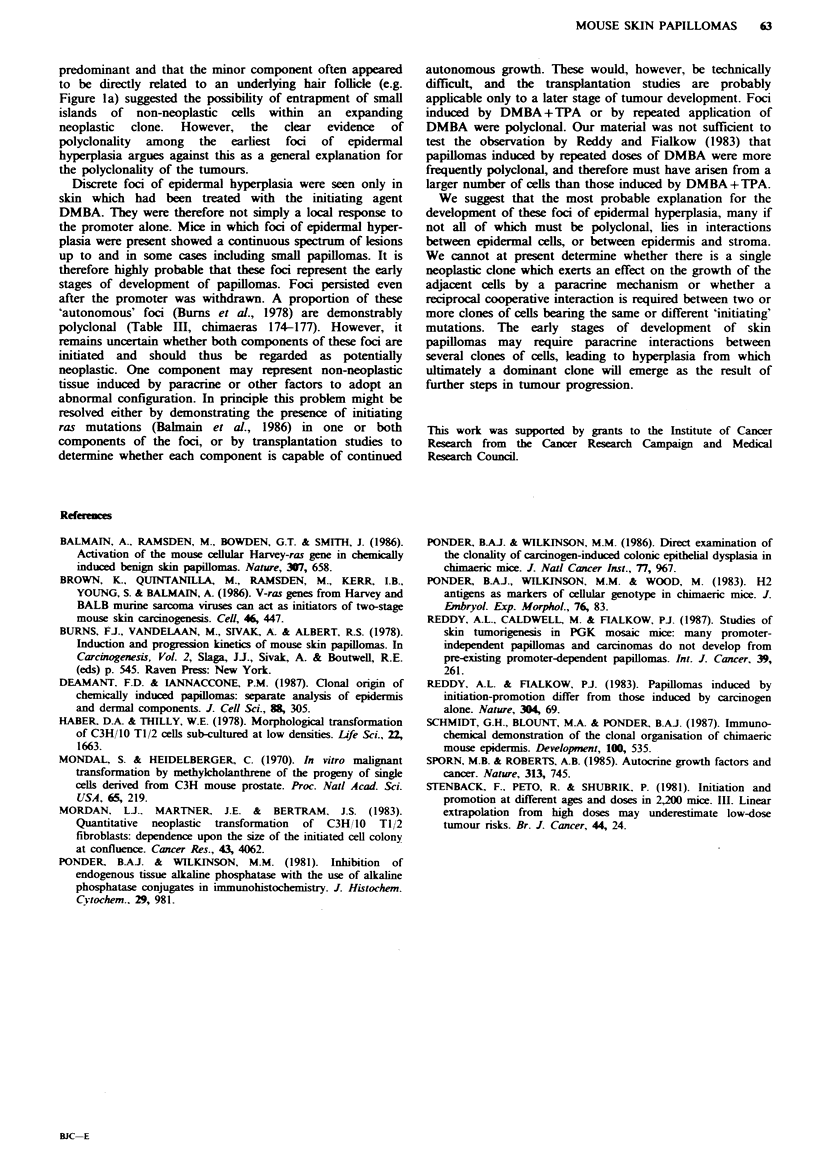# Polyclonal origin of mouse skin papillomas.

**DOI:** 10.1038/bjc.1989.220

**Published:** 1989-07

**Authors:** D. J. Winton, M. A. Blount, B. A. Ponder

**Affiliations:** Institute of Cancer Research, Haddow Laboratories, Sutton, Surrey, UK.

## Abstract

**Images:**


Br. J. Cancer (1989), 60, 59-63 © The Macmillan Press Ltd., 1989

### Polyclonal origin of mouse skin papillomas

D.J. Winton, M.A. Blount & B.A.J. Ponder

*Institute of Cancer Research, Haddow Laboratories, Cotswold Road, Sutton, Surrey SM2 5NG, UK.*

**Summary** We show that, from the earliest morphologically recognisable stages of development, mouse skin papillomas induced by a chemical initiation-promotion regime are polyclonal. We have demonstrated polyclonality directly by immunohistochemical staining of the mosaic cell populations in embryo aggregation chimaeras, a method which removes some of the uncertainties of previous conclusions based on analysis using electrophoretic polymorphisms. The findings may imply that initiation within a single cell and promotion of its clonal descendents is not a sufficient explanation for the origin of these tumours and that interaction between cells of more than one clone is involved.

Current theories of multistage carcinogenesis, in which emphasis is placed on a somatic mutation as the initiating event, predict the origin of tumours from a single cell. Progression to malignancy occurs by subsequent changes in that initiated cell and its progeny. Analysis of human tumours and of tumours induced experimentally in animals has for the most part shown them to be clonal, consistent with this prediction. However, many tumours have been sampled at a relatively advanced stage when clonal selection may have occurred, and some reports suggest polyclonality, so the possibility remains that some tumours may be of multicellular origin. Multicellular origin would imply that interactions between cells, or between a group of cells and the local environment, are important in tumorigenesis. Evidence that tumour cells may produce both autocrine and paracrine growth-stimulating substances has provided a possible mechanism for such interaction (Sporn & Roberts, 1985). In addition, there is evidence from both *in vivo* and *in vitro* experiments that initiation may be a more frequent event than transformation (Mondal & Heidelberger, 1970; Stenback *et al.*, 1981); and that transformation of initiated cells in culture can be inhibited by co-culture with an excess of non-initiated cells (Haber & Thilly, 1978; Mordan *et al.*, 1983). Both of these observations could be explained by mechanisms involving interactions between initiated cells and their neighbours.

Most analyses of the clonal composition of tumours have used electrophoretic polymorphisms as clonal markers, either in X-chromosome mosaics or in mouse embryo aggregation chimaeras. The interpretation of the electrophoretic studies presents two major problems. To be sampled, a tumour must be large enough to be visible and dissected away from normal tissue, which means it will contain many tens of thousands of cells. Monoclonality at this stage may not reflect the origin of the tumour, but the later selection of a dominant clone. Conversely, polyclonal composition may be due to contamination of the tumour sample by non-neoplastic tissue: because the sample is destroyed in the analysis, this can never be directly determined.

Problems of this kind may explain the continuing conflict of evidence about the clonal composition of chemically induced mouse skin papillomas, which are variously suggested to arise from one or from two or more cells (Reddy & Fialkow, 1983; Reddy *et al.*, 1987, Deamant & Iannaccone, 1987). To overcome these problems, we have made an immunohistochemcial study of the clonal composition of skin papillomas in chimaeric mice. The mice are constructed by aggregation of embryos of CBA/Ca (CBA) and C57BL.Cbi (C57BL) strains. The tissues of these mice are a mosaic of cells derived from each of the 'parent' embryos. We have previously described the demonstration of the mosaicism in normal tissues of these mice, including epidermis, using immunohistochemistry with monoclonal antibodies to H2 specificities (CBA mice are of $H2^k$ haplotype, and C57BL mice $H2^b$) (Schmidt *et al.*, 1987; Ponder *et al.*, 1983); and we have applied this technique to the clonal analysis of colonic dysplasia (Ponder & Wilkinson, 1986). Here we show unequivocally that the epidermal component of some papillomas and of the earliest foci of epidermal hyperplasia (EFH) which precede papilloma formation is polyclonal.

### Materials and methods

### *Mice*

CBA/Ca and C57BL/6J mice were obtained from OLAC (Bicester, UK) at 6-8 weeks of age. C57BL.Cbi mice were bred at the Institute of Cancer Research. Embryo aggregation chimaeras were constructed as previously described (Ponder *et al.*, 1983). Mice were maintained on SMC dust-free grade 6 softwood sawdust (Sawdust Marketing Co., Standon, Herts., UK). Water and SDS no. 1 (modified) expanded diet (Special Diet Services Ltd, Witham, Essex, UK) were supplied *ad libitum*.

### *Carcinogenesis*

Mice were shaved 72 h before application of initiator and only mice showing no hair regrowth were used. Initiation was with 200 μg or 400 μg dimethylbenzanthracene (DMBA: Aldrich) dissolved in 0.2 ml Aristar grade acetone (BDH, Poole, UK) and applied to a 2 × 4 cm area of shaved dorsal skin. Mice not receiving DMBA were treated with acetone only. Promotion was with 12-*O*-tetradecanoyl phorbol-13-acetate (TPA: Sigma, Poole, UK) administered 10 μg twice weekly in 200 μl acetone, commencing 1 week after initiation, or with repeated once weekly application of 40 μg DMBA.

### *Papillomas*

Control and chimaeric mice were examined at each treatment for the presence of papillomas. At 3-4 week intervals, selected tumours of approximately 0.5 mm or more in diameter were excised with a collar of adjacent skin, and TPA treatment was withheld for 1 week. Tumours were mounted in OCT (Tissue-Tek: Miles Laboratories, Naperville, Ill., USA) and snap frozen for immunohistochemical analysis.

### *Epidermal focal hyperplasia (EFH)*

Groups of CBA and C57BL.Cbi mice were initiated with 200 μg DMBA or mock-initiated with acetone, and promoted with TPA or DMBA as shown in Tables I and II respectively. At predetermined time points (Tables I and II),

Correspondence: B.A.J. Ponder.
Received 14 February 1989, and accepted in revised form 21 March 1989.

**Table I** Incidence of EFH in initiated and uninitiated mice treated with TPA

| Treatment | | | | | Mean scores per mouse[a] | | | | | |
|---|---|---|---|---|---|---|---|---|---|---|
| | | | Time | | Normal | | | Abnormal | | |
| Init. | Prom. (weeks) | Mouse strain[b] | killed (weeks) | No. mice | 1 | 2 | 3 | 4 | 5 | P[c] |
| DMBA | 10 | C57BL | 12 | 6 | 4.7 | 1.8 | 1.8 | 1.5 | 0.2 | 0 |
| – | 10 | C57BL | 12 | 4 | 7.2 | 2.8 | 0 | 0 | 0 | 0 |
| DMBA | 10 | CBA | 12 | 5 | 5.6 | 2.6 | 1.8 | 0 | 0 | 0 |
| – | 10 | CBA | 12 | 4 | 8.2 | 1.5 | 0.2 | 0 | 0 | 0 |
| DMBA | 14 | C57BL | 16 | 6 | 4.3 | 2.0 | 1.7 | 0.5 | 0.7 | 0.8 |
| – | 14 | C57BL | 16 | 4 | 10.0 | 0 | 0 | 0 | 0 | 0 |
| DMBA | 14 | CBA | 16 | 6 | 6.9 | 1.5 | 0.7 | 0.3 | 0.2 | 0.5 |
| – | 14 | CBA | 16 | 4 | 9.2 | 0.8 | 0 | 0 | 0 | 0 |
| DMBA | 18 | C57BL | 30 | 4 | 5.0 | 1.2 | 1.8 | 0.2 | 0 | 1.8 |
| DMBA | 18 | CBA | 30 | 8 | 6.5 | 2.1 | 0.6 | 0.1 | 0 | 0.6 |

[a]Ten samples per mouse. Only the most advanced lesion in each sample was scored. EFH were graded 1–5 or papillomas as defined in Materials and methods. The number of EFH of each category were totalled for each treatment and these divided by the number of mice scored; [b]C57BL.Cbi and CBA/Ca strains; [c]$P$ = papilloma.

**Table II** Incidence of EFH in DMBA-initiated mice promoted with DMBA

| | | | | Mean scores per mouse[a] | | | | | |
|---|---|---|---|---|---|---|---|---|---|
| | | Time | | Normal | | | Abnormal | | |
| Treatment | Mouse strain[b] | killed (weeks) | No. mice | 1 | 2 | 3 | 4 | 5 | P[c] |
| DMBA/DMBA-10w[d] | C57BL | 12 | 3 | 9.0 | 0.7 | 0.3 | 0 | 0 | 0 |
| | CBA | 12 | 6 | 7.8 | 1.3 | 0.7 | 0 | 0 | 0.2 |
| DMBA/DMBA-14w | C57BL | 16 | 3 | 9.3 | 0.3 | 0 | 0 | 0.3 | 0 |
| | CBA | 16 | 6 | 7.5 | 1.7 | 0.8 | 0 | 0 | 0 |
| DMBA/DMBA-23w | C57BL | 25 | 5 | 4.9 | 1.4 | 1.4 | 0.9 | 0.5 | 0.9 |
| | CBA | 25 | 5 | 4.6 | 2.2 | 2.2 | 0.8 | 0 | 0.2 |

[a]Ten samples per mouse. Only the most advanced lesion in each sample was scored. EFH were graded 1–5 or papilloma as defined in Materials and methods. The number of EFH of each category were totalled for each treatment and these divided by the numbers of mice scored; [b]C57BL.Cbi and CBA/Ca strains; [c]$P$ = papilloma; [d]Mice initiated with 200 μg DMBA and promoted with 40 μg DMBA per week for 10 weeks.

mice were taken at random, killed by cervical dislocation, and the dorsal and flank skin was removed in one piece, mounted on card and fixed in 10% formol saline. The treated skin was sampled by cutting out 10 full-thickness strips each 13 × 2 mm running parallel to the spine in a standard pattern. These were processed by standard methods and embedded on edge in three paraffin blocks for histology. Four μm sections were taken of each block at each of five levels about 200 μm apart, stained with Haematoxylin and Eosin, coded and scored for evidence of foci of epidermal hyperplasia without knowledge of their origin. A section from the middle level of each block was scored first, and each lesion identified was noted and sought on the adjacent levels (mounted on the same slide) to ensure that so far as possible the most advanced area of the lesion was scored. Foci were arbitrarily graded 1–5 in increasing degrees of abnormality: 1 = completely normal; 2 = borderline abnormality; 3 = small focus with epidermis at least five cells thick, extending over a length of 50–100 cells in the plane of section; 4 = larger focus with epidermis at least five cells thick, extending over 100–200 cells, dermis normal; 5 = large focus, as 4 but with beginning elevation of the epidermal lesion and distortion of underlying dermis. Lesions with marked elevation were classifed as papilloma. To avoid problems over the independence of adjacent lesions, only the single most advanced lesion was scored in each sample.

Groups of chimaeric mice were treated in parallel (Table III) but 6 weeks behind, so that the results from the CBA and C57Bl.Cbi non-chimaeric mice would indicate the best time to analyse the chimaeras for foci of epidermal hyperplasia. Chimaeras were killed and the dorsal skin sampled as above although the strips were slightly smaller (approximately 2 × 10 mm) and were mounted on edge unfixed in OCT for cryostat sectioning. Consecutive 6 μm sections were cut at each of five levels approximately 200 μm apart: a representative section from the middle level of each block was stained for H2 antigens (see below), scored for foci of hyperplasia, and the corresponding sections from adjacent levels stained and examined to confirm the limited extent of any lesions, as above.

*Immunohistochemical staining*

The mouse monoclonal antibodies 11.4-1 (anti $H2^k$) and FT6α9 (anti $H2^b$) were used as direct conjugates to alkaline phosphatase and to peroxidase respectively, as previously described (Ponder et al., 1983). Alkaline phosphatase was demonstrated using naphthol AS-BI sodium salt (Sigma) coupled to Brentamine Fast Red TR in veronal acetate buffer pH 9.2, giving a red reaction product. Peroxidase was demonstrated using 3′3′ diaminobenzidine (Sigma) in 50 mM tris pH 7.2 as substrate, giving a brown reaction product. Endogenous alkaline phosphatase was inhibited by 1% levamisole in the substrate solution (Ponder & Wilkinson, 1981), and peroxidase by pretreatment of the sections with 0.1% phenyl hydrazine hydrochloride. Sections were counterstained with haemalum. Non-chimaeric CBA and C57BL skin sections were stained with both antibody conjugates simultaneously and separately in parallel with chimaeric skin samples in every staining run, to provide positive and negative staining controls.

### Results

### *Papillomas induced by DMBA + TPA*

*Control mice* The sensitivity of CBA and C57BL mice to the initiation–promotion regime was confirmed in pilot studies. In the first experiment, eight CBA/Ca and eight C57BL/6J mice were initiated with 400 μg DMBA and promoted with TPA 10 μg twice weekly. These mice developed 12 and 16 papillomas over 0.5 mm diameter respectively (average 1.5 and 2.0 per mouse) by 18 weeks of treatment. These tumours were excised for immunohistochemical staining for H2 antigens (see below). Supply problems necessitated a switch from C57BL/6J to C57BL.Cbi mice: because of this a second pilot study was done using eight CBA/Ca and eight C57BL.Cbi mice, and an initiating dose of 200 μg DMBA. After 18 weeks of TPA twice weekly, these groups of mice had developed respectively 15 and 31 papillomas >0.5 mm in diameter: an average of 1.9 and 3.9 tumours per mouse.

Immunohistochemical staining of papillomas from these mice for H2 antigens showed that while H2 staining was occasionally weak or focally absent in larger papillomas, in 14 CBA and 10 C57BL papillomas in which clear staining for the appropriate H2 antigen was obtained, there was no evidence of staining with antibody to the inappropriate H2 haplotype.

*Chimaeras* Eight CBA/Ca↔C57BL.Cbi chimaeras were initiated with 200 μg DMBA and promoted with 10 μg TPA twice weekly for 24 weeks. A total of 43 papillomas (0.5–5 mm diameter) were removed as they developed, with a small collar of adjacent epidermis. Twelve of 43 tumours (28%) contained both $H2^b$ and $H2^k$ epidermal cells within the tumour mass as determined by simultaneous staining with the two antibody conjugates (Figures 1a and 2a); in a further five (12%), the tumour itself was composed of cells of the same H2 type, but patches of the opposite H2 type were present in the hyperplastic epidermis bordering the tumour. In each of the 'mixed' tumours, one component predominated. Frequently, the minor component appeared to correspond to the position of hair follicles of that H2 type in the dermis underlying the tumour, raising the possibility that the minor component might in some cases represent entrapped normal cells (Figure 1a).

To examine the origin of the polyclonality of these tumours in more detail, a study was made of the earliest focal hyperplastic lesions which could be detected following initiation and promotion.

### *Epidermal focal hyperplasia*

Table I shows that focal areas of epidermal hyperplasia (defined as epidermis at least five cells thick; for examples, see Figure 2b–d) developed within 10 weeks of starting TPA promotion in CBA and C57BL.Cbi non-chimaeric mice which had been initiated with DMBA, but were not seen in the absence of initiation. Some lesions were still present 12 weeks after discontinuing TPA treatment, which indicates that they had developed autonomy from continued promotion. As with the development of papillomas, CBA appeared to be less sensitive than C57BL.Cbi mice. Mice promoted with low doses (40 μg week$^{-1}$) of DMBA were slower to develop areas of EFH but these were apparent after 23 weeks of treatment (Table II).

The clonal composition of similar foci of hyperplasia was examined in 11 CBA↔C57BL.Cbi chimaeras treated according to three different regimes. The results are summarised in Table III and examples shown in Figures 1 and 2. Approximately 20% of lesions were of mixed phenotype in each of the three experimental groups. In the majority of lesions (e.g. Figures 1d and 2d) there was a well defined boundary between hyperplastic and normal epidermis; in others, no clear boundary could be seen.

### *Mosaic patch sizes in normal epidermis*

In each of the CBA↔C57BL.Cbi chimaeras examined, the C57BL.Cbi ($H2^b$) component formed the majority of the epidermis, with the result that in tissue sections large $H2^b$ patches were interspersed with infrequent small patches of $H2^k$ epidermis. Analysis in tissue sections of a 10 mm length of epidermis chosen at random from each of the 11 chimaeras showed that there were on average 0.88 patch boundaries per millimetre (equivalent to approximately one boundary per 100 cells). Because of the variation in $H2^b$:$H2^k$ proportions from chimaera to chimaera and the irregular distribution of mosaic patches within the epidermis (Schmidt *et al*., 1987), the frequency of patch boundaries will have been very variable both between and within individual chimaeric mice.

### Discussion

Immunohistochemical staining shows clearly that the epidermal component of about 25% of chemically induced mouse skin papillomas is polyclonal. This confirms the suggestion of polyclonality by Reddy & Fialkow (1983) (where the possibility of contamination by non-neoplastic tissue left some doubt), and evidence for polyclonality based on viral integration sites in papillomas induced by epidermal innoculation of Harvey murine sarcoma virus (Brown *et al*., 1986).

That most of the tumours and hyperplasias we studied were of a single H2 phenotype does not of course imply that they were monoclonal. A polyclonal tumour will only have a mixed phenotype if it arises across a boundary between mosaic patches in the chimaeric epidermis. In the CBA↔C57BL.Cbi chimaeras used in these experiments, our data suggest that on average there was a boundary every 100 cells. Twenty per cent foci of epidermal hyperplasia with mixed phenotype is therefore compatible with the origin of these foci from regions of the epidermis measuring very approximately 20 cells in diameter.

Papillomas and foci of hyperplasia were too infrequent for the polyclonality to be explained as the result of coalescence of independently-arising neoplastic clones. The observation that in all the mixed papillomas one component was

**Table III** Clonal composition of focal epidermal hyperplasia in chimaeric mice

| *Chimaeras* | *Treatment* | *Total no. of foci scored* | *No. of foci of mixed phenotype* |
|---|---|---|---|
| 168–170 | DMBA → TPA 9–11 weeks sampled 2 weeks from last dose of TPA | 40 | 8 (20%) |
| 174–177 | DMBA → TPA 20 weeks sampled week 24 | 30 | 5 (16.7%) |
| 178–181 | DMBA → DMBA 22 weeks sampled week 24 | 21 | 5 (24%) |
| | | | 18/91 = 19.8% |

**Figure 1** H-2 double stain of chimaeric epidermis from mice treated with DMBA/TPA or DMBA/DMBA. Adjacent H&E sections are shown in Figure 2. Cryostat sections (6 μm) counterstained with haemalum. C57BL (H-$2^b$) tissue is stained yellow-brown and CBA (H-$2^k$) is stained red. All illustrations taken from mice initiated with 200 μg DMBA. **a**, Mixed papilloma, mainly H-$2^b$ (brown) but with a minor H-$2^k$ (red) component, excised after 18 weeks TPA promotion. Note 'entrapment' of CBA component arising from hair follicle (arrow) ( × 100). **b**, Large mixed focus of epidermal hyperplasia (EFH). 11 weeks TPA promotion, sampled 2 weeks after promotion stopped ( × 120). **c**, Small polyclonal EFH. 18 weeks TPA promotion, sampled 4 weeks after promotion stopped ( × 320). **d**, Part of the EFH shown in Figure 2d, to illustrate the well defined boundary (arrow) to the hyperplastic epidermis, and polyclonal composition. Promoted for 20 weeks with DMBA (40 μg week$^{-1}$); sampled 2 weeks after promotion stopped ( × 290).

**Figure 2** Adjacent H&E stained sections for comparison with Figure 1. In **b**, **c** and **d** the area corresponding to that shown in Figure 1 is indicated by arrows. **a**, Papilloma ( × 100). **b**, Large area of TPA-induced EFH ( × 70). **c**, Small area of TPA-induced EFH ( × 200). **d**, DMBA-induced EFH ( × 120).

predominant and that the minor component often appeared to be directly related to an underlying hair follicle (e.g. Figure 1a) suggested the possibility of entrapment of small islands of non-neoplastic cells within an expanding neoplastic clone. However, the clear evidence of polyclonality among the earliest foci of epidermal hyperplasia argues against this as a general explanation for the polyclonality of the tumours.

Discrete foci of epidermal hyperplasia were seen only in skin which had been treated with the initiating agent DMBA. They were therefore not simply a local response to the promoter alone. Mice in which foci of epidermal hyperplasia were present showed a continuous spectrum of lesions up to and in some cases including small papillomas. It is therefore highly probable that these foci represent the early stages of development of papillomas. Foci persisted even after the promoter was withdrawn. A proportion of these 'autonomous' foci (Burns *et al.*, 1978) are demonstrably polyclonal (Table III, chimaeras 174–177). However, it remains uncertain whether both components of these foci are initiated and should thus be regarded as potentially neoplastic. One component may represent non-neoplastic tissue induced by paracrine or other factors to adopt an abnormal configuration. In principle this problem might be resolved either by demonstrating the presence of initiating *ras* mutations (Balmain *et al.*, 1986) in one or both components of the foci, or by transplantation studies to determine whether each component is capable of continued autonomous growth. These would, however, be technically difficult, and the transplantation studies are probably applicable only to a later stage of tumour development. Foci induced by DMBA+TPA or by repeated application of DMBA were polyclonal. Our material was not sufficient to test the observation by Reddy and Fialkow (1983) that papillomas induced by repeated doses of DMBA were more frequently polyclonal, and therefore must have arisen from a larger number of cells than those induced by DMBA+TPA.

We suggest that the most probable explanation for the development of these foci of epidermal hyperplasia, many if not all of which must be polyclonal, lies in interactions between epidermal cells, or between epidermis and stroma. We cannot at present determine whether there is a single neoplastic clone which exerts an effect on the growth of the adjacent cells by a paracrine mechanism or whether a reciprocal cooperative interaction is required between two or more clones of cells bearing the same or different 'initiating' mutations. The early stages of development of skin papillomas may require paracrine interactions between several clones of cells, leading to hyperplasia from which ultimately a dominant clone will emerge as the result of further steps in tumour progression.

This work was supported by grants to the Institute of Cancer Research from the Cancer Research Campaign and Medical Research Council.

### References

BALMAIN, A., RAMSDEN, M., BOWDEN, G.T. & SMITH, J. (1986). Activation of the mouse cellular Harvey-*ras* gene in chemically induced benign skin papillomas. *Nature*, **307**, 658.

BROWN, K., QUINTANILLA, M., RAMSDEN, M., KERR, I.B., YOUNG, S. & BALMAIN, A. (1986). V-*ras* genes from Harvey and BALB murine sarcoma viruses can act as initiators of two-stage mouse skin carcinogenesis. *Cell*, **46**, 447.

BURNS, F.J., VANDELAAN, M., SIVAK, A. & ALBERT, R.S. (1978). Induction and progression kinetics of mouse skin papillomas. In *Carcinogenesis, Vol. 2*, Slaga, J.J., Sivak, A. & Boutwell, R.E. (eds) p. 545. Raven Press: New York.

DEAMANT, F.D. & IANNACCONE, P.M. (1987). Clonal origin of chemically induced papillomas: separate analysis of epidermis and dermal components. *J. Cell Sci.*, **88**, 305.

HABER, D.A. & THILLY, W.E. (1978). Morphological transformation of C3H/10 T1/2 cells sub-cultured at low densities. *Life Sci.*, **22**, 1663.

MONDAL, S. & HEIDELBERGER, C. (1970). *In vitro* malignant transformation by methylcholanthrene of the progeny of single cells derived from C3H mouse prostate. *Proc. Natl Acad. Sci. USA*, **65**, 219.

MORDAN, L.J., MARTNER, J.E. & BERTRAM, J.S. (1983). Quantitative neoplastic transformation of C3H/10 T1/2 fibroblasts: dependence upon the size of the initiated cell colony at confluence. *Cancer Res.*, **43**, 4062.

PONDER, B.A.J. & WILKINSON, M.M. (1981). Inhibition of endogenous tissue alkaline phosphatase with the use of alkaline phosphatase conjugates in immunohistochemistry. *J. Histochem. Cytochem.*, **29**, 981.

PONDER, B.A.J. & WILKINSON, M.M. (1986). Direct examination of the clonality of carcinogen-induced colonic epithelial dysplasia in chimaeric mice. *J. Natl Cancer Inst.*, **77**, 967.

PONDER, B.A.J., WILKINSON, M.M. & WOOD, M. (1983). H2 antigens as markers of cellular genotype in chimaeric mice. *J. Embryol. Exp. Morphol.*, **76**, 83.

REDDY, A.L., CALDWELL, M. & FIALKOW, P.J. (1987). Studies of skin tumorigenesis in PGK mosaic mice: many promoter-independent papillomas and carcinomas do not develop from pre-existing promoter-dependent papillomas. *Int. J. Cancer*, **39**, 261.

REDDY, A.L. & FIALKOW, P.J. (1983). Papillomas induced by initiation-promotion differ from those induced by carcinogen alone. *Nature*, **304**, 69.

SCHMIDT, G.H., BLOUNT, M.A. & PONDER, B.A.J. (1987). Immunochemical demonstration of the clonal organisation of chimaeric mouse epidermis. *Development*, **100**, 535.

SPORN, M.B. & ROBERTS, A.B. (1985). Autocrine growth factors and cancer. *Nature*, **313**, 745.

STENBACK, F., PETO, R. & SHUBRIK, P. (1981). Initiation and promotion at different ages and doses in 2,200 mice. III. Linear extrapolation from high doses may underestimate low-dose tumour risks. *Br. J. Cancer*, **44**, 24.